# Supplementary material for: Pharmacologic IRE1/XBP1s activation promotes systemic adaptive remodeling in obesity
Source: Nat Commun. 2022 Feb 1;13:608. doi: 10.1038/s41467-022-28271-2 (PMC8807832; doi:10.1038/s41467-022-28271-2)
Supplement: Supplementary file 1 — Supplementary Information [file 41467_2022_28271_MOESM1_ESM.pdf]

Supplementary Information for:

Pharmacologic IRE1/XBP1s Activation Promotes Systemic Adaptive Remodeling in Obesity

Aparajita Madhavan[1,4], Bernard P. Kok[1,4], Bibiana Rius[1], Julia M.D. Grandjean[1], Adekunle Alabi[1], Verena Albert[1], Ara Sukiasyan[1], Evan T. Powers[2], Andrea Galmozzi[1,3], Enrique Saez[1]*, R. Luke Wiseman[1]*

[1]Department of Molecular Medicine, The Scripps Research Institute, La Jolla, CA 92037
[2]Department of Chemistry, The Scripps Research Institute, La Jolla, CA 92037
[3]Department of Medicine, University of Wisconsin, Madison, WI 53705

[4]These authors contributed equally

*To whom correspondence should be addressed:

Enrique Saez
Department of Molecular Medicine
The Scripps Research Institute
La Jolla, CA 92037
Email: esaez@scripps.edu

R. Luke Wiseman
Department of Molecular Medicine
The Scripps Research Institute
La Jolla, CA 92037
Email: wiseman@scripps.edu

Running Title: IXA4 improves systemic metabolism in obesity

Key Words: unfolded protein response; proteostasis; insulin signaling; glucose homeostasis; obesity; gluconeogenesis; insulin secretion

SUPPLEMENTARY INFORMATION TABLE OF CONTENTS

Supplementary Information Cover Page…………....………………………………………………………………..1

Supplementary Information Table of Contents………….……………………………………………………..2

Fig. S1: IXA4 is a selective activator of protective IRE1/XBP1s signaling in liver……………………………3

Fig. S2: Effects of IXA4 treatment on weight, food intake, and insulin signaling in muscle and liver…….5

Fig. S3: Impact of IXA4 treatment on liver gluconeogenesis and lipogenesis in DIO mice…………………6

Fig. S4: IXA4 activates IRE1/XBP1s signaling in Min6 cells………………………………………………………7

Supplementary Data Legends……….…………….……………………………………….……………………..8

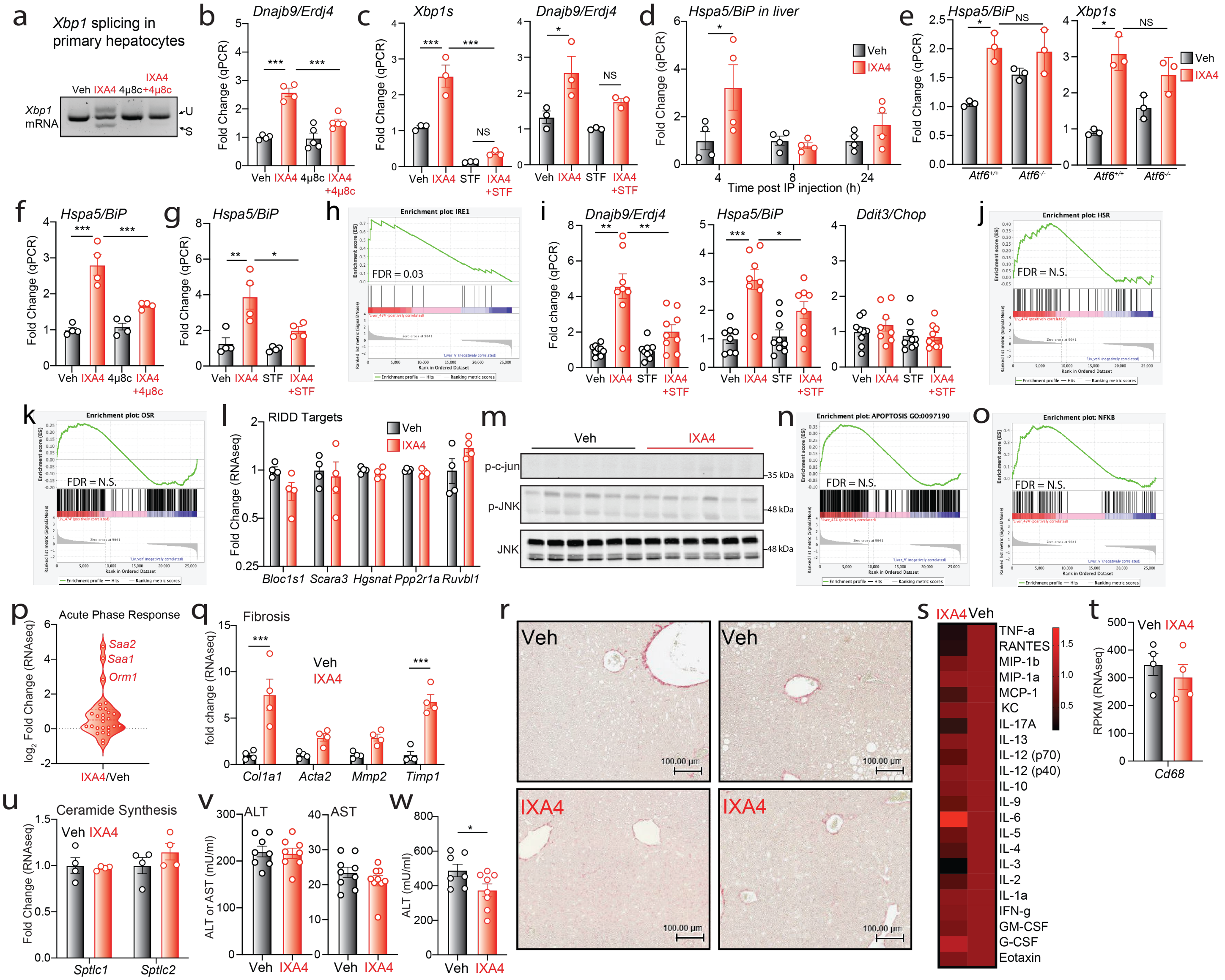

Fig. S1: IXA4 is a selective activator of protective IRE1/XBP1s signaling in liver

a-c, *Xbp1* splicing (**a**) and expression, measured by qPCR of *Xbp1s* and the IRE1 target gene *Dnajb9* in primary mouse hepatocytes treated for 12 h with IXA4 (10 μM) and/or the IRE1 RNase inhibitors 4μ8c (32 μM) or STF-083010 (STF; 64 μM). Error bars show SEM for n=3-5 replicates. *p<0.05,***p<0.001 for one-way ANOVA. **d,** Expression, measured by RT-qPCR, of the UPR target gene *Hspa5/BiP* in liver of chow-fed mice 4, 8, or 24 h after IP injection of IXA4 50 mg/kg). Error bars show SEM for n=4 mice/condition. *p<0.05 for two-way ANOVA. **e,** Expression, measured by RT-qPCR, of *Hspa5/BiP* and *Xbp1s* in *Atf6*$^{+/+}$ and *Atf6*$^{-/-}$ MEFs treated for 12 h with vehicle or IXA4 (10 μM). Error bars show SEM for n=3. *p<0.05 for one-way ANOVA. **f,** Expression, measured by RT-qPCR, of *Hspa5/BiP* in primary hepatocytes treated for 12 h with IXA4 (10 μM) and/or 4μ8c (32 μM). Error bars show SEM for n=4. ***p<0.001 for one-way ANOVA. **g,** Expression, measured by RT-qPCR of *Hspa5* in liver of mice 4 h after IP dosing with IXA4 (50 mg/kg) and/or STF-083010 (STF, 10 mg/kg). Error bars show SEM for n=4 mice/condition. *p<0.05 for one-way ANOVA. **h,** GSEA performed on RNA-seq data from livers of DIO mice treated with IXA4 for 8 weeks for IRE1 signaling. **i,** Expression, measured by RT-qPCR, of *Dnajb9*, *Hspa5*, and *Ddit3* in liver of DIO mice treated for 21 days with IXA4 and/or STF-083010 (STF; 5 mg/kg). Error bars show SEM for n=8 or 9 mice/condition. *p<0.05, **p<0.01, ***p<0.001 for a two-tailed Welch's t-test. **j,k,** GSEA performed on RNAseq data from livers of DIO mice treated with IXA4 for 8 weeks for the heat shock response (HSR) and the oxidative stress response (OSR). **l,** Expression, measured by RNAseq, of RIDD targets in liver of DIO mice after 8 weeks of IXA4 treatment. Error bars show SEM for n=4 mice/condition. **m,** Immunoblot of c-jun and JNK phosphorylation in liver of DIO mice after 8 weeks of IXA4 treatment. **n,o,** GSEA performed on RNAseq data from livers of DIO mice treated with IXA4 for 8 weeks for apoptosis (**n**) and NFκB (**o**). **p,q,** Fold change,

assessed with RNAseq, of the acute phase response geneset (**p**) and fibrosis genes (**q**) in livers of DIO mice treated with IXA4 for 8 weeks. ***p<0.01 for two-way ANOVA. **r,** Representative images of Sirius red staining of DIO mouse livers after 8 weeks of IXA4 treatment. **s,** Heat map of average levels of plasma cytokines (n=5-9 mice/condition; mice where cytokines fell below the range of detection were excluded from this analysis) in IXA4-treated DIO mice normalized to vehicle. **t,u,** Expression, assessed with RNAseq, of *Cd68* (**t**) and ceramide synthesis genes (**u**) in liver of DIO mice treated with IXA4 for 8 weeks. Error bars show SEM for n=4 mice/condition **v,w,** Plasma alanine transaminase (ALT) and aspartate transaminase (AST) levels in DIO mice treated with IXA4 for 3 (**v**) or 8 (**w**) weeks. Error bars show SEM for n=7 or 8 mice/condition. *p<0.05 for a two-tailed Welch's t-test. Source data for all panels in this figure are provided as **Source Data File S1**. Uncropped images of the immunoblots in panel (**m**) are included in **Source Data**.

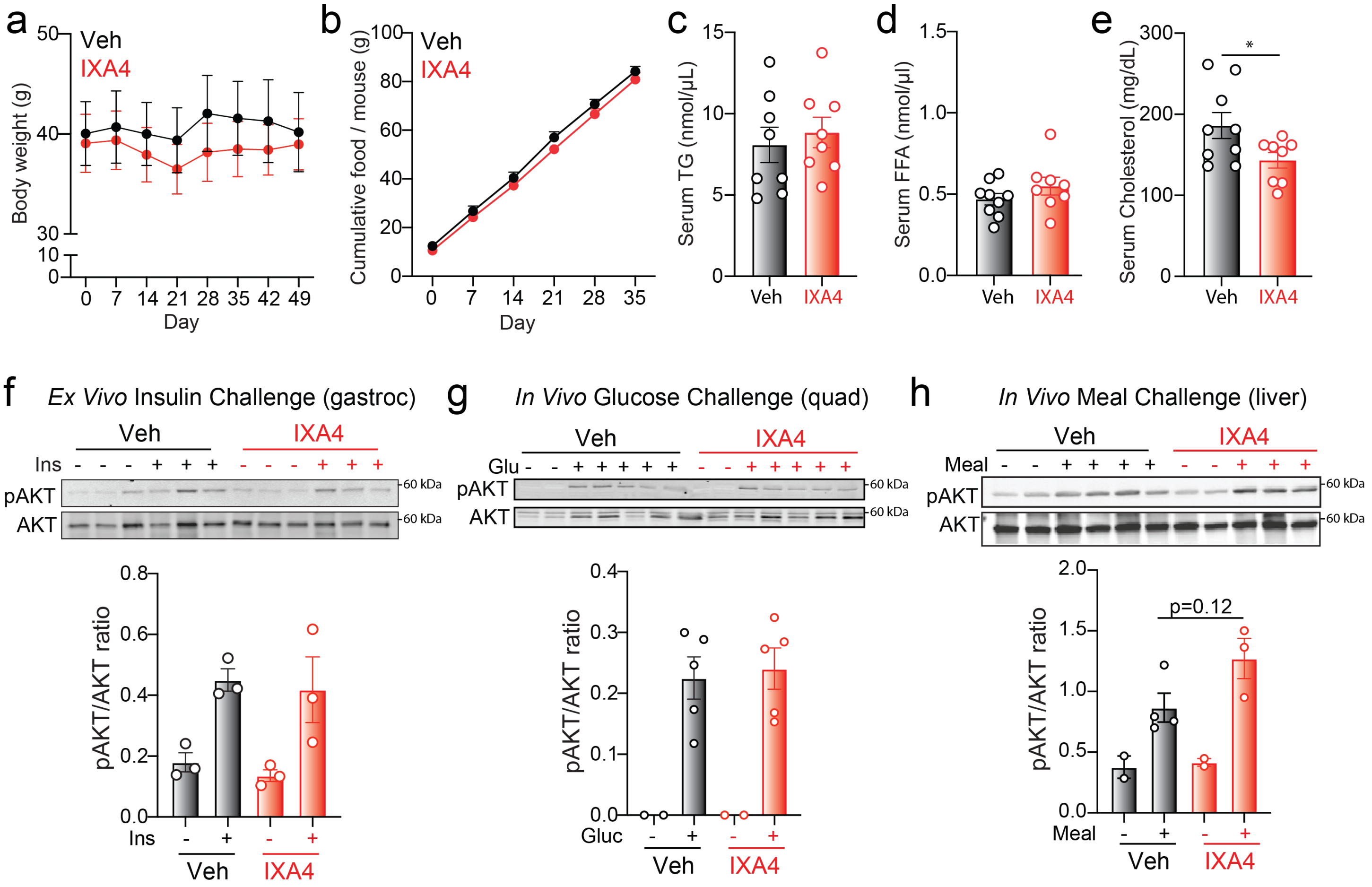

Fig. S2: Effects of IXA4 treatment on weight, food intake, and insulin signaling in muscle and liver
a,b, Body weight (**a**) and food intake (**b**) over time for vehicle and IXA4-treated DIO mice. **c-e**, Plasma levels of triglycerides (**c**), free fatty acids (**d**), and cholesterol (**e**) in DIO treated with vehicle or IXA4. Error bars show SEM for n=8 or 9 mice/condition. *p<0.05 for a two-tailed Welch's t-test. **f,** Immunoblot and quantification of pAKT/AKT ratio in gastrocnemius muscle isolated from IXA4 or vehicle treated DIO mice and stimulated *ex vivo* with insulin (10 nM) for 10 min. Error bars show SEM for n=3. **g,** Immunoblot and quantification of pAKT/AKT ratio in quadricep muscle of DIO mice treated for 60 days with IXA4 or vehicle, 15 min after oral glucose administration. Error bars show SEM for n=2 or 5. **h,** Immunoblot and quantification of pAKT/AKT ratio in liver of DIO mice treated for 62 days with IXA4 or vehicle, 15 min after oral administration of a complex meal (Ensure). Error bars show SEM for n=2-4 replicates. p-value from a two-tailed Welch's t-test. Source data for all panels in this figure are provided as **Source Data File S2**. Uncropped images of the immunoblots in panels (**f**), (**g**), and (**h**), are included in **Source Data**.

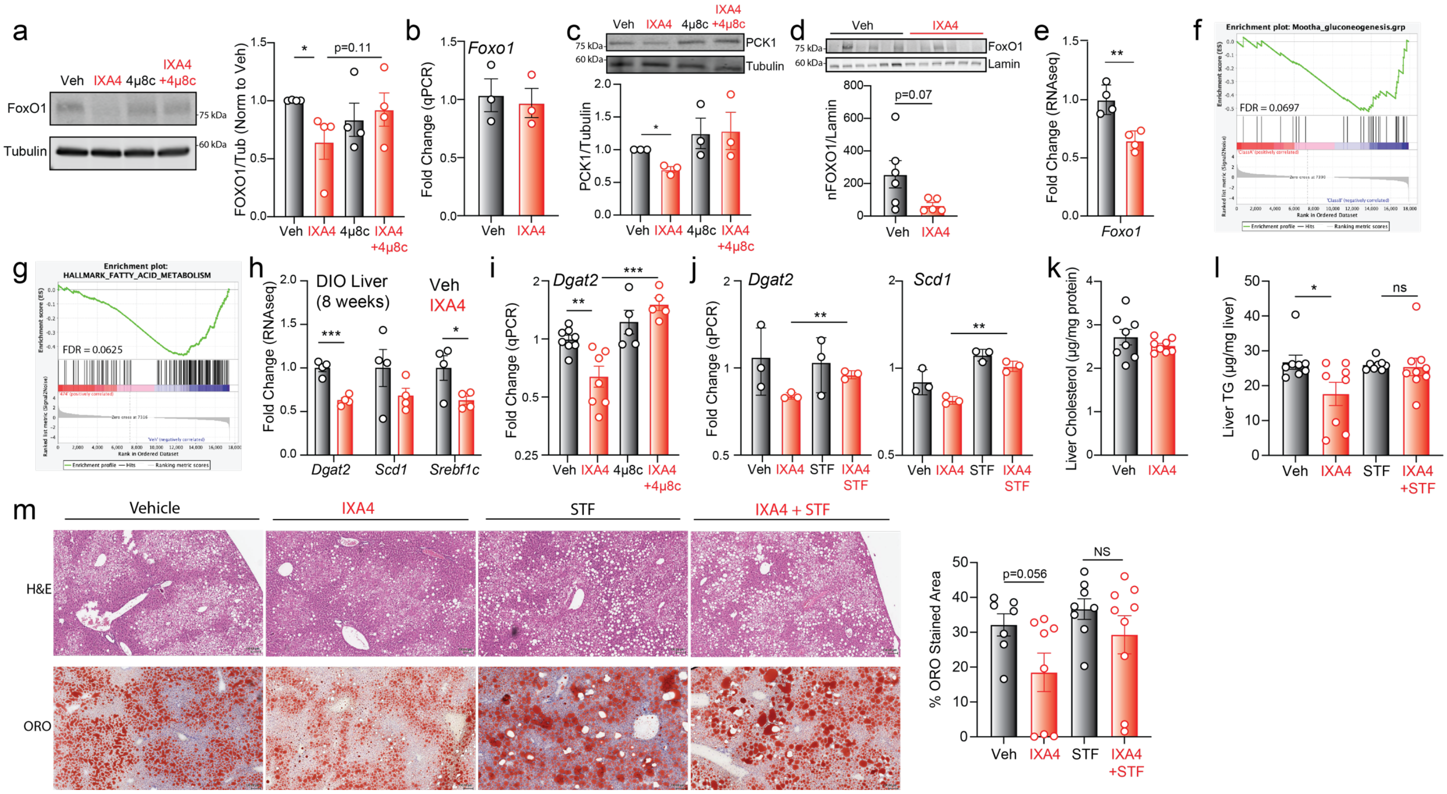

Fig. S3: Impact of IXA4 treatment on liver gluconeogenesis and lipogenesis in DIO mice

a, Immunoblot and quantification of total FOXO1 protein and tubulin in primary mouse hepatocytes treated for 12 h with IXA4 (10 μM) and/or the IRE1 RNase inhibitor 4μ8c (32 μM). Error bars show SEM for n=4. *p<0.05 for a two-tailed Welch's t-test. **b,** *Foxo1* mRNA levels after 12 h of treatment with IXA4 (10 μM) and/or 4μ8c (32 μM) in primary mouse hepatocytes. Error bars show SEM for n=3 replicates. **c,** Immunoblot and quantification of PCK1 in primary mouse hepatocytes treated for 12 h with IXA4 (10 μM) and/or 4μ8c (32 μM). Error bars show SEM n=3. *p<0.05 for one-way ANOVA. **d,** Immunoblot and quantification of FOXO1 protein in nuclear fractions prepared from liver of vehicle and IXA4-treated DIO mice after 8 weeks. Error bars show SEM for n=5 or 6 mice/condition. p-value for a two-tailed Welch's t-test is shown. One outlier identified by the ROUT outlier test was removed from this analysis. **e,** Fold change in *Foxo1* mRNA measured with RNAseq in liver of DIO mice treated with IXA4 for 8 weeks. Error bars show SEM for n=4 mice/condition. *p<0.05 from a two-tailed Welch's t-test. **f,g,** GSEA analysis of liver RNA-seq data of DIO mice treated with IXA4 for 8 weeks for gluconeogenesis (**f**) and fatty acid metabolism (**g**). **h,** Fold change, assessed using RNAseq, of lipogenic genes in liver of DIO mice treated with IXA4 for 8 weeks. Error bars show SEM for n=4 mice/condition. *p<0.05, ***p<0.001 for a two-tailed Welch's t-test. **i,j,** Expression, measured by qPCR, of lipogenic genes in mouse primary hepatocytes treated for 12 h with IXA4 (10 μM) and/or either of the IRE1 inhibitors 4μ8c (32 μM) or STF-083010 (STF; 64 μM). Error bars show SEM for n=3-7, as indicated. *p<0.05, **p<0.01, ***p<0.001 for a two-tailed Welch's t-test. **k,** Cholesterol content in liver of DIO mice treated with IXA4 for 8 weeks. Error bars show SEM for n=8 mice/condition. **l,** Triglyceride content in liver of DIO mice treated with IXA4 for 3 weeks. Error bars show SEM for n=8 or 9 mice/condition. *p<0.05 for a two-tailed Welch's t-test. **m,** Representative liver images and quantification of DIO mice treated with IXA4 and/or STF-083010 (STF; 5 mg/kg) for 3 weeks, stained with H&E or oil red O (ORO). Quantification of area stained with ORO is shown. Error bars show SEM for n = 7-9 individual images. p-values for a two-tailed Welch's t-test are shown. Source data for all panels in this figure are provided as **Source Data File S3**. Uncropped images of the immunoblots in panels (**a**), (**c**), and (**d**) are included in **Source Data**.

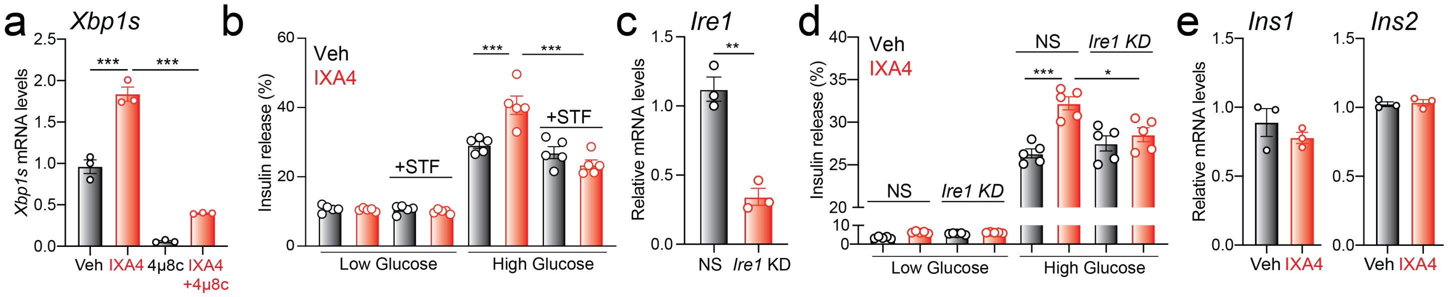

Fig. S4: IXA4 activates IRE1/XBP1s signaling in Min6 cells

a, Expression, measured by RT-qPCR, of *Xbp1s* in Min6 cells after a 6 h treatment with IXA4 (10 μM) and/or the IRE1 inhibitor 4μ8c (32 μM). Error bars show SEM for n=3 replicates. ***p<0.001 for one-way ANOVA. **b,** Insulin release from Min6 cells pretreated for 36 h with vehicle, IXA4 (10 μM), and/or STF-083010 (STF; 32 μM) and then stimulated with media containing low (2.8 mM) or high (16.8 mM) glucose for 60 min. Error bars show SEM for n=5 replicates. ***p<0.001 for one-way ANOVA. **c,** Expression, measured by RT-qPCR, of *Ire1* in Min6 cells cells infected with lentivirus expressing non-silencing (NS) or *Ire1* shRNA. Error bars show SEM for n=3 replicates. **p<0.01 for a two-tailed Welch's t-test. **d,** Insulin release from Min6 cells expressing non-silencing (NS) or *Ire1* shRNA pretreated for 36 h with vehicle and/or IXA4 (10 μM) and then stimulated with media containing low (2.8 mM) or high (16.8 mM) glucose for 60 min. Error bars show SEM for n=5 replicates. *p<0.05, ***p<0.001 for a two-tailed Welch's t-test. **e,** Expression, measured by RT-qPCR, of *Ins1* and *Ins2* mRNA in Min6 cells after a 6 h treatment with IXA4 (10 μM). Error bars show SEM for n=3 replicates. Source data for all panels in this figure are provided as **Source Data File S4**.

SUPPLEMENTARY DATA LEGENDS

Supplementary Data 1. Differential expression (DESeq) analysis of RNA-seq data from liver of DIO mice treated with IXA4 relative to vehicle-treated mice.

Supplementary Data 2. Expression (measured using RNA-seq) of UPR target genes primarily regulated downstream of ATF6, IRE1/XBP1s, or PERK signaling.

Supplementary Data 3. Gene ontology analysis of differentially expressed transcripts (RNA-seq) in livers of DIO mice treated with IXA4 relative to vehicle-treated mice.

Supplementary Data 4. Fold change expression of gluconeogenesis genes (RNA-seq) in livers of DIO mice treated with IXA4 relative to vehicle-treated mice.
