# Supplementary material for: Pharmacologic IRE1/XBP1s activation promotes systemic adaptive remodeling in obesity
Source: Nat Commun. 2022 Feb 1;13:608. doi: 10.1038/s41467-022-28271-2 (PMC8807832; doi:10.1038/s41467-022-28271-2)
Supplement: Supplementary file 3 — Description of Additional Supplementary Files [file 41467_2022_28271_MOESM3_ESM.docx]

Description of Additional Supplementary Files

Title: Supplementary Data 1.

Description: Differential expression (DESeq) analysis of RNA-seq data from liver of DIO mice treated with IXA4 relative to vehicle-treated mice.

Title: Supplementary Data 2.

Description: Expression (measured using RNA-seq) of UPR target genes primarily regulated downstream of ATF6, IRE1/XBP1s, or PERK signaling.

Title: Supplementary Data 3.

Description: Gene ontology analysis of differentially expressed transcripts (RNA-seq) in livers of DIO mice treated with IXA4 relative to vehicle-treated mice.

Title: Supplementary Data 4.

Description: Fold change expression of gluconeogenesis genes (RNA-seq) in livers of DIO mice treated with IXA4 relative to vehicle-treated mice.
