# Supplementary material for: Pharmacologic IRE1/XBP1s activation promotes systemic adaptive remodeling in obesity
Source: Nat Commun. 2022 Feb 1;13:608. doi: 10.1038/s41467-022-28271-2 (PMC8807832; doi:10.1038/s41467-022-28271-2)
Supplement: Supplementary file 8 — Reporting Summary [file 41467_2022_28271_MOESM8_ESM.pdf]

nature research

Corresponding author(s): R. Luke Wiseman, Enrique Saez

Last updated by author(s): Jan 8, 2022

Reporting Summary

Nature Research wishes to improve the reproducibility of the work that we publish. This form provides structure for consistency and transparency in reporting. For further information on Nature Research policies, see our Editorial Policies and the Editorial Policy Checklist.

Statistics

For all statistical analyses, confirm that the following items are present in the figure legend, table legend, main text, or Methods section.

n/a	Confirmed	
☐	☒	The exact sample size (n) for each experimental group/condition, given as a discrete number and unit of measurement
☐	☒	A statement on whether measurements were taken from distinct samples or whether the same sample was measured repeatedly
☐	☒	The statistical test(s) used AND whether they are one- or two-sided<br>*Only common tests should be described solely by name; describe more complex techniques in the Methods section.*
☐	☒	A description of all covariates tested
☐	☒	A description of any assumptions or corrections, such as tests of normality and adjustment for multiple comparisons
☐	☒	A full description of the statistical parameters including central tendency (e.g. means) or other basic estimates (e.g. regression coefficient) AND variation (e.g. standard deviation) or associated estimates of uncertainty (e.g. confidence intervals)
☐	☒	For null hypothesis testing, the test statistic (e.g. F, t, r) with confidence intervals, effect sizes, degrees of freedom and P value noted<br>*Give P values as exact values whenever suitable.*
☒	☐	For Bayesian analysis, information on the choice of priors and Markov chain Monte Carlo settings
☒	☐	For hierarchical and complex designs, identification of the appropriate level for tests and full reporting of outcomes
☒	☐	Estimates of effect sizes (e.g. Cohen's d, Pearson's r), indicating how they were calculated

Our web collection on statistics for biologists contains articles on many of the points above.

Software and code

Policy information about availability of computer code

Data collection	DNBseq (BGI proprietary RNA-seq platform); Licor Imaging Studios
Data analysis	Excel 16.21, GraphPad Prism 9.3.0, ImageJ, DNAstar Lasergene SeqManPro, ArrayStar 12.2, R, Panther (geneontology.org), GSEA.4.0.3, ImageJ. Code for standard open-source DESeq differential gene expression RNAseq analysis used in R statistical software is available from the corresponding authors upon reasonable request.

For manuscripts utilizing custom algorithms or software that are central to the research but not yet described in published literature, software must be made available to editors and reviewers. We strongly encourage code deposition in a community repository (e.g. GitHub). See the Nature Research guidelines for submitting code & software for further information.

Data

Policy information about availability of data

All manuscripts must include a data availability statement. This statement should provide the following information, where applicable:

- Accession codes, unique identifiers, or web links for publicly available datasets
- A list of figures that have associated raw data
- A description of any restrictions on data availability

The raw data that support the findings of this study are available as Source Data included in this manuscript. The RNA-seq data is deposited to the public National Center for Biotechnology Information GEO repository (GSE162567).

Field-specific reporting

Please select the one below that is the best fit for your research. If you are not sure, read the appropriate sections before making your selection.

[x] Life sciences [ ] Behavioural & social sciences [ ] Ecological, evolutionary & environmental sciences

For a reference copy of the document with all sections, see nature.com/documents/nr-reporting-summary-flat.pdf

Life sciences study design

All studies must disclose on these points even when the disclosure is negative.

Sample size	Samples sizes for each experiment are shown as individual points. Sample size was determined by power analysis.
Data exclusions	Outliers were identified using the ROUT outlier test in PRISM and removed from the reported analysis because they were not representative of the experimental outcomes. Specific figures where outliers were omitted are indicated in the figure legend.
Replication	Major findings reported in this manuscript were confirmed across two independent cohorts.
Randomization	DIO mice were assigned to different treatment groups based on body weight and fasting glucose to minimize differences in body weight and fasting glucose across groups prior to the start of chronic treatment.
Blinding	Mice and samples were not blinded to the experimenters because the experimenters had to dose mice with specific treatments.

Reporting for specific materials, systems and methods

We require information from authors about some types of materials, experimental systems and methods used in many studies. Here, indicate whether each material, system or method listed is relevant to your study. If you are not sure if a list item applies to your research, read the appropriate section before selecting a response.

Materials & experimental systems

n/a	Involved in the study	
[ ]	[x]	Antibodies
[ ]	[x]	Eukaryotic cell lines
[x]	[ ]	Palaeontology and archaeology
[ ]	[x]	Animals and other organisms
[x]	[ ]	Human research participants
[x]	[ ]	Clinical data
[x]	[ ]	Dual use research of concern

Methods

n/a	Involved in the study	
[x]	[ ]	ChIP-seq
[x]	[ ]	Flow cytometry
[x]	[ ]	MRI-based neuroimaging

Antibodies

Antibodies used	Antibodies used in this study include: phospho-c-jun (Cell Signaling, catalog no. 3270S; 1:1000), phospho-JNK (Cell Signaling, catalog no. 4668S; 1:1000), JNK (Cell Signaling, catalog no. 9252S; 1:1000), phospho-AKT(Ser473) (Cell Signaling, catalog no. 4060S: 1:2000), AKT (Cell Signaling, catalog no. 2920S; 1:1000), FOXO1 (Cell Signaling, catalog no. 2880S; 1:1000), LaminB1 (Cell Signaling, catalog no. 13435S; 1:1000), tubulin (Sigma, catalog no. T6074-200UL; 1:5000), XBP1s(E9V3E) (Cell Signaling, catalog no. 40435S; 1:500), BiP (Cell Signaling, catalog no. 3177S; 1:1000), SEC24D (gift from William Balch's lab at Scripps; 1:1000), PERK (Cell Signaling, catalog no 3192S; 1:1000), eIF2 (Cell Signaling, catalog no 9722; 1:1000), phospho-eIF2 (Cell Signaling, catalog no 97211: 1000), and PCK1 (Abcam, catalog no. ab703581; 1000).
Validation	Antibody validation is reported on the manufacturers website that can be freely accessed using the detailed information provided above.

Eukaryotic cell lines

Policy information about cell lines

Cell line source(s)	Min6 cells were a kind gift from Maike Sander at UCSD and ATF6+/+ and ATF6-/- MEFs were a kind gift from Randal Kaufman at Sanford Burnham
Authentication	None of the cells were authenticated.
Mycoplasma contamination	Cells are routinely tested for mycoplasma every 3-4 months. All cells were mycoplasma negative.

Commonly misidentified lines (See ICLAC register)	We did not use any misidentified cell lines.

Animals and other organisms

Policy information about studies involving animals; ARRIVE guidelines recommended for reporting animal research

Laboratory animals	C57BL/6 male mice. All mice were between 14 and 25 weeks of age.
Wild animals	No wild animals were used in this study.
Field-collected samples	No field collected samples were used in this study.
Ethics oversight	All procedures were approved by the Scripps Research IACUC.

Note that full information on the approval of the study protocol must also be provided in the manuscript.
