# Supplementary material for: Pharmacologic IRE1/XBP1s activation promotes systemic adaptive remodeling in obesity
Source: Nat Commun. 2022 Feb 1;13:608. doi: 10.1038/s41467-022-28271-2 (PMC8807832; doi:10.1038/s41467-022-28271-2)

Fig. 1g. Uncropped Gels

Xbp1s mRNA splicing gel

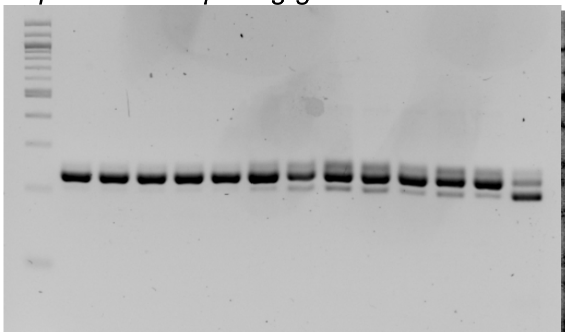

XBP1s Protein Gel

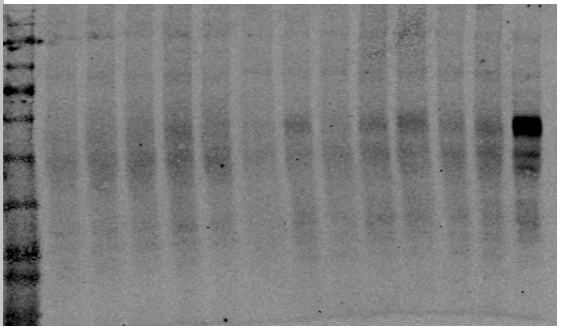

BIP Protein gel

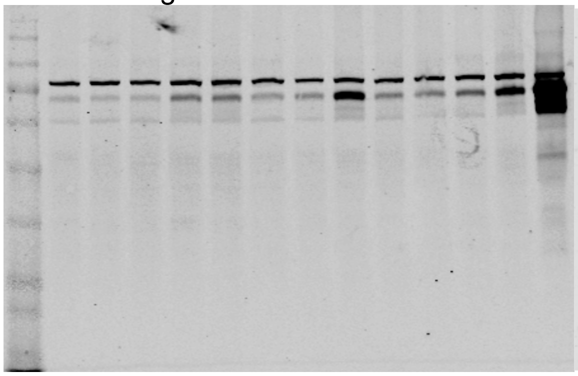

SEC24D Protein Gel

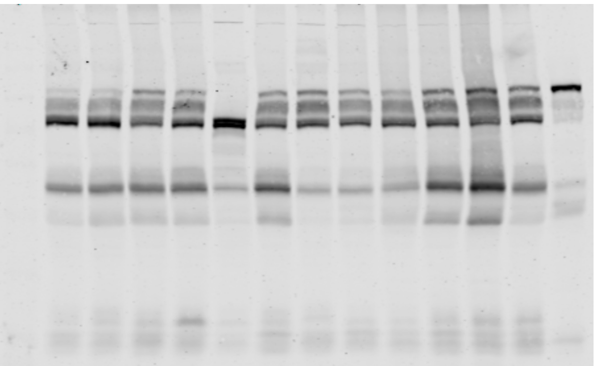

PERK Protein Gel

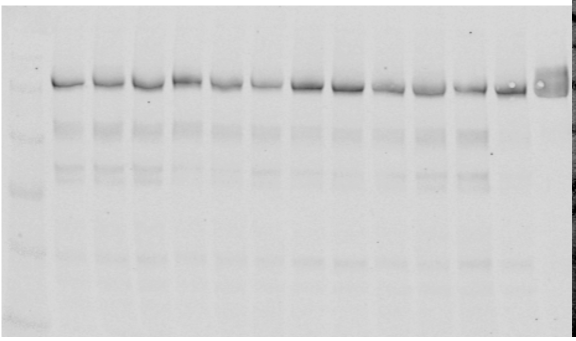

p-eIF2α Protein Gel

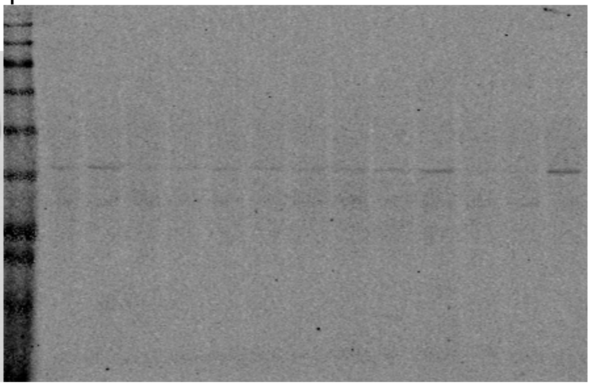

Total eIF2α Protein Gel

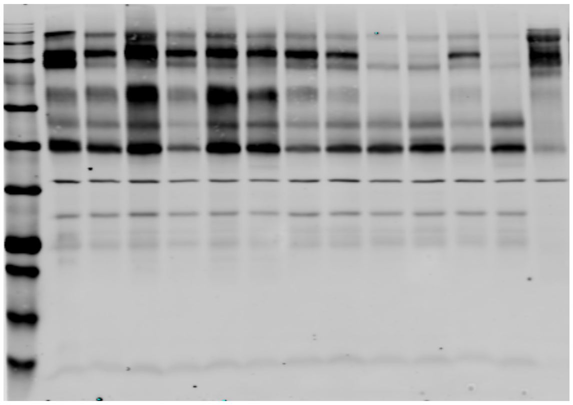

Fig. 2j Uncropped Gels

p-AKT Protein Blot

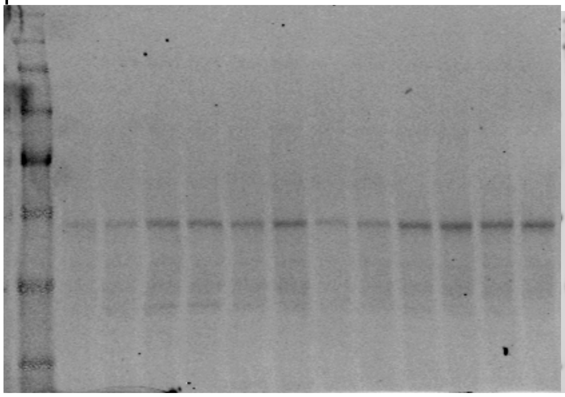

Total AKT Protein Blot

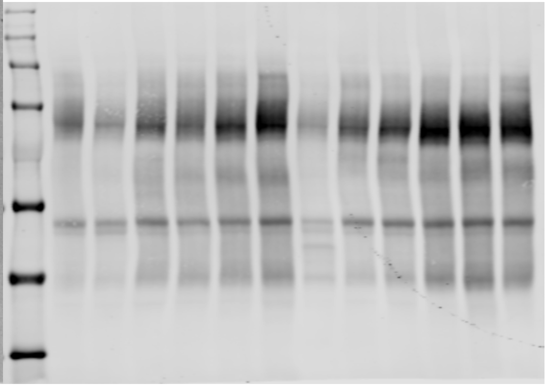

Fig. S2m Uncropped Gels

p-JNK Protein Blot

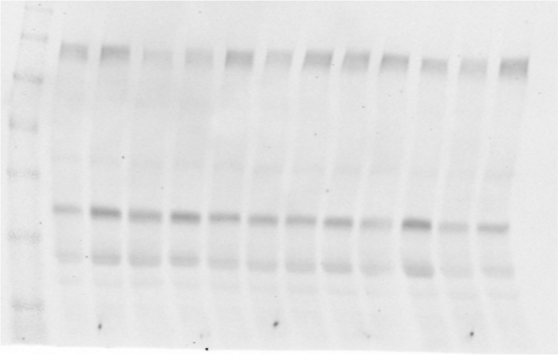

JNK Protein Blot

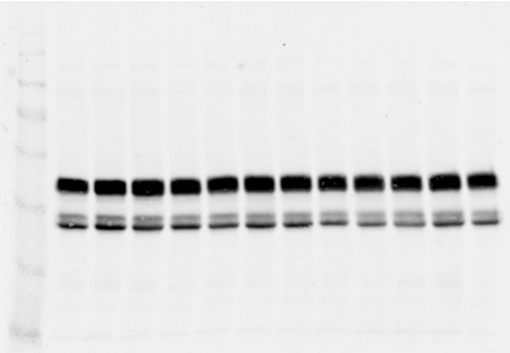

Fig. S2f Uncropped Gels

pAKT

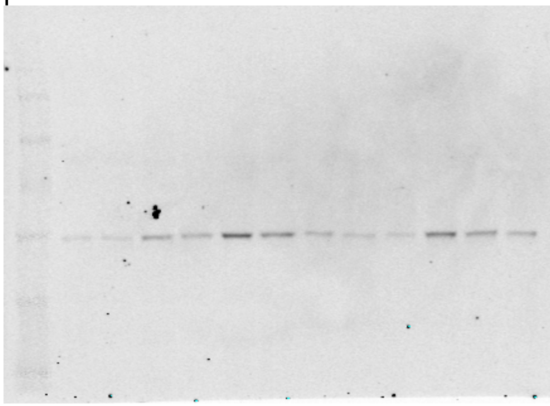

Total AKT

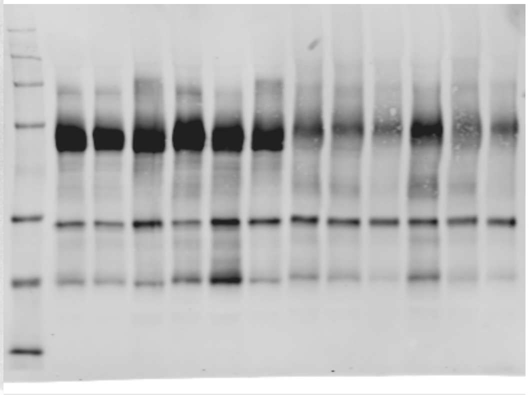

Fig. S2g Uncropped Gels

pAKT

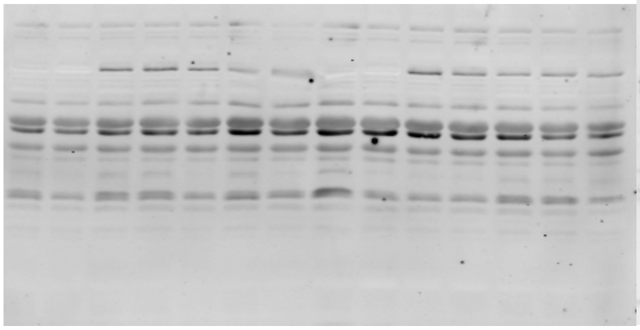

Total AKT

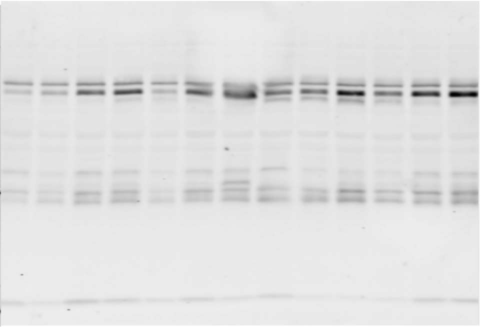

Fig. S2h Uncropped Gels

pAKT

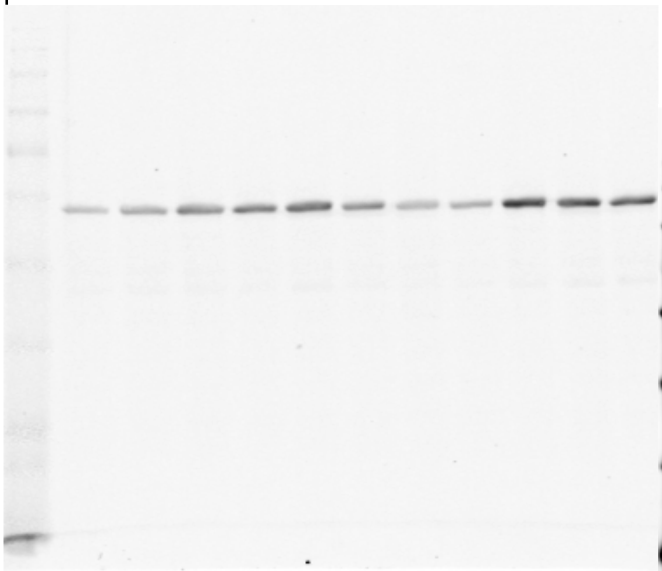

Total AKT

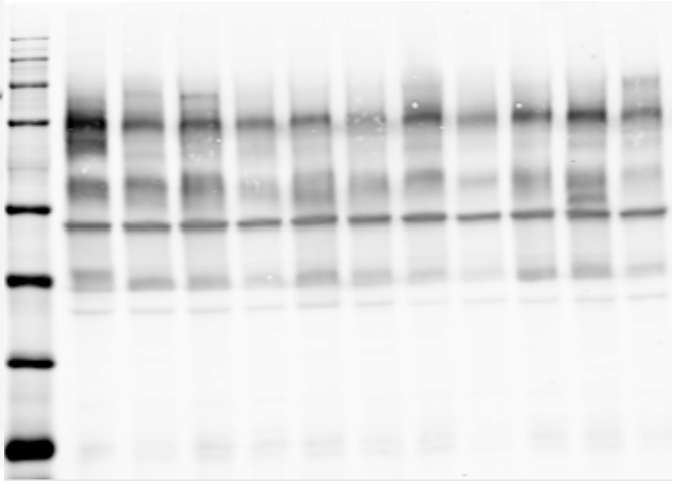

Fig. S3a Uncropped Gels

FOXO1

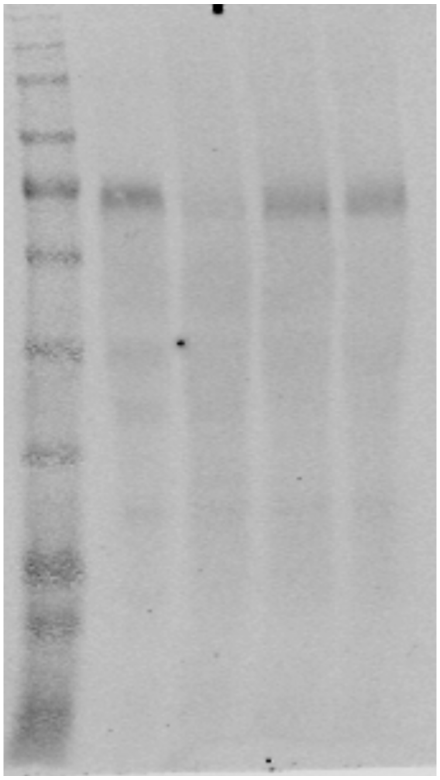

Tubulin

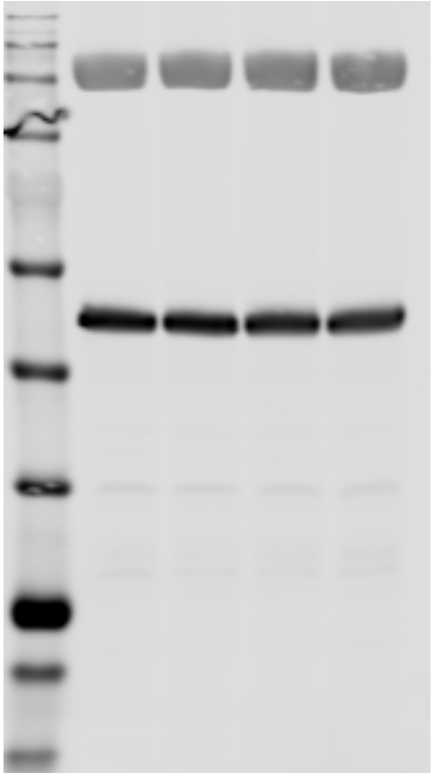

Fig. S3c Uncropped Gels

PCK1

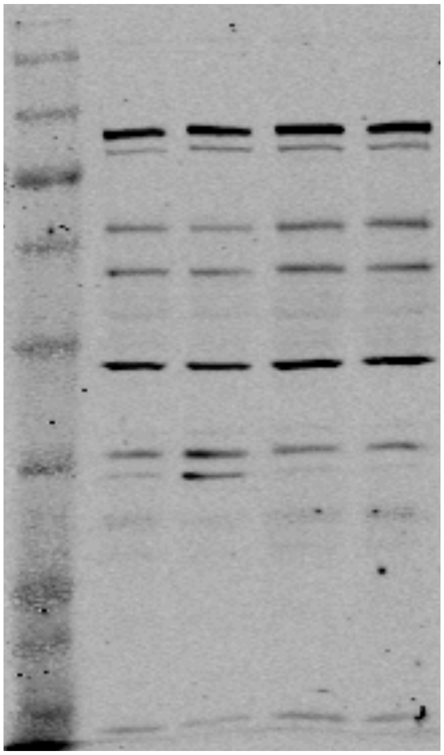

Tubulin

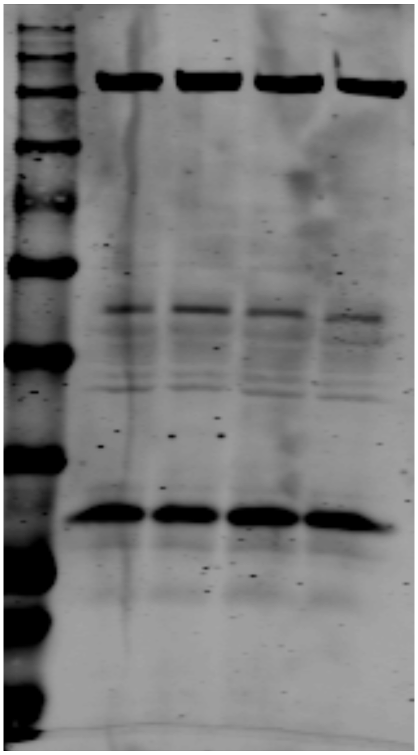

Fig. S3d Uncropped Gels

FOXO1

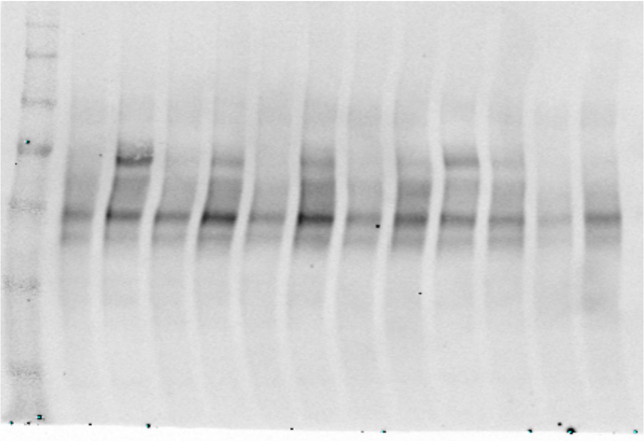

Lamin

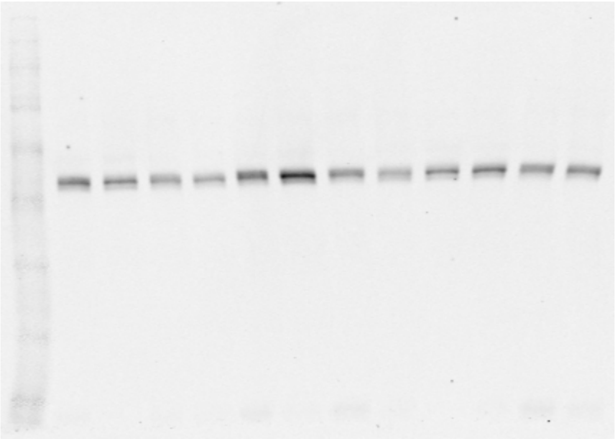

Supplement: Supplementary file 9 — Source Data [file 41467_2022_28271_MOESM9_ESM.zip › Source Data/Source Data Uncropped Gels.pdf]
